# Detecting a periodic signal by a population of spiking neurons in the weakly nonlinear response regime

**DOI:** 10.1140/epje/s10189-023-00371-x

**Published:** 2023-11-06

**Authors:** Maria Schlungbaum, Benjamin Lindner

**Affiliations:** 1 Physics Department, Humboldt University Berlin, Berlin, Germany; 2 Bernstein Center for Computational Neuroscience Berlin, Berlin, Germany

## Abstract

**Abstract:**

Motivated by experimental observations, we investigate a variant of the cocktail party problem: the detection of a weak periodic stimulus in the presence of fluctuations and another periodic stimulus which is stronger than the periodic signal to be detected. Specifically, we study the response of a population of stochastic leaky integrate-and-fire (LIF) neurons to two periodic signals and focus in particular on the question, whether the presence of one of the stimuli can be detected from the population activity. As a detection criterion, we use a simple threshold-crossing of the population activity over a certain time window. We show by means of the receiver operating characteristics (ROC) that the detectability depends only weakly on the time window of observation but rather strongly on the stimulus amplitude. Counterintuitively, the detection of the weak periodic signal can be facilitated by the presence of a strong periodic input current depending on the frequencies of the two signals and on the dynamical regime in which the neurons operate. Beside numerical simulations of the model, we present an analytical approximation for the ROC curve that is based on the weakly nonlinear response theory for a stochastic LIF neuron.

**Graphic abstract:**

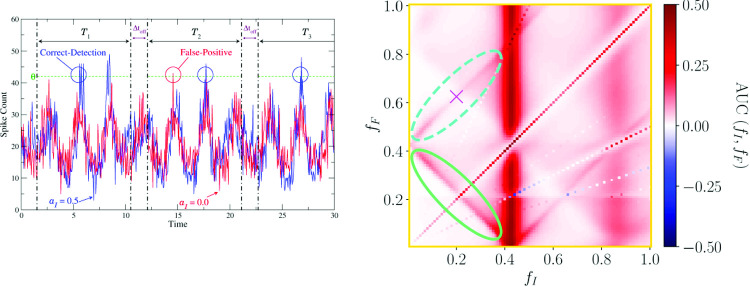

## Introduction

The detection of a weak signal in the presence of a much stronger signal is an interesting problem that arises in several natural situations for living organisms, most prominently in auditory perception where it is known as the cocktail party problem [1]. Detection is complicated by nonlinearity in the sensory apparatus (see e.g. [2]) and by different noise sources [3], so studying very simple models can help us to better understand this problem.

The basic problem has been thoroughly investigated in the stochastic processes community with respect to beneficial effects that noise can have in nonlinear dynamical systems. It is well-known that the detection and transmission of a weak signal can be improved by a finite amount of fluctuations by the mechanism of stochastic resonance as reviewed and critically discussed in Refs. [4–8]. Different stochastic resonance effects have been shown to occur in theoretical models like point processes [9, 10], simple threshold systems [11–15], bistable [16–24], and excitable systems [25–30]. This theoretical research was stimulated by experimental demonstrations of stochastic resonance in electronic circuits [31], laser systems [32], ion channels in biological membranes [33], neural receptors [34–36], and spatially extended neural systems [37, 38] to name but a few examples. More recent contributions in the context of neuroscience discuss in particular the role of the intrinsic noise in neural systems [39–43] and their interplay with abundant mechanisms of adaptation in neural systems [44–46]. Generally, how neural populations of spiking neurons respond to time-dependent stimuli has been addressed with different theoretical approaches [43, 47–52].

An intriguing example of a signal detection task can be found in the courtship behavior of weakly electric fish. It has been observed that a resident male is able to detect a distant male intruder while courting a female [53]. This represents an instance of the aforementioned cocktail party problem: a comparatively weak low-frequency signal (the distant intruder) has to be detected in the presence of another time-dependent input, coming from the nearby female, a strong high-frequency stimulus. The detection of this faint signal is a fascinating problem that involves many levels of neural processing and is additionally complicated by the movements of the participating fish [53], by the change of their frequencies (known as jamming avoidance response) [54], and by different adaptation mechanisms (starting with a pronounced spike-frequency adaptation in the sensory receptor cells, the P-units) [55].

Here, we take the specific experimental observation of an intruder detection as an inspiration to study a generic detection problem of how one periodic signal can be detected in the presence of another one by a population of stochastically spiking neurons. The generic scheme may also be applicable to other sensory modalities in which concurring periodic signals are present (the sense of hearing would be an obvious case). Our interest lies in the first stage of signal processing (neglecting possible filters or optimized detectors on higher levels), where we analyze how different time-dependent stimuli are present in the output of uncoupled noisy cells. We would like to stress that our simple detection scheme is not supposed to account for the observed detection performance in weakly electric fish. (None of the above-mentioned complications is taken into account.) However, to the best of our knowledge, even this simple model, that allows for some analytical insights, has not been studied yet. The key questions that we will be interested in here are: Under which conditions is the periodic signal easier to detect—in the presence or the absence of the second (strong periodic) background stimulus? Which role is played by the stochastic firing regime of the neurons in the population (mean- or fluctuation-driven regime) and by the response regime (linear or weakly nonlinear)? Are there specific frequency combinations of the two stimuli that make the weaker signal better detectable?

Assuming a simple detection scheme based on spike counts, we want to explore the roles of linear and nonlinear responses in the stochastic dynamics of the single cell. We hypothesize that the weakly nonlinear response may play a beneficial role in the detection task and that also the presence of the strong periodic background stimulus (i.e., the female courtship input in the above example of weakly electric fish) does not have to be necessarily detrimental for the detection but, on the contrary, may facilitate the detection. We would like to emphasize that this beneficial effect is unrelated to the *vibrational* resonance effect emerging in systems driven by multiple periodic signals (see discussion below).

Our paper is organized as follows: First, we introduce the model and sketch how we perform the measurement process and how the detector functions. We then present our analytical approximation of the receiver operating characteristic (ROC) which quantifies the detection performance. We investigate the influence of variations of the simulation and detection parameters in the detectability of the periodic signal and focus in particular on the detection time window, the strength of the signal amplitude, and the frequency combinations. We consider two operating regimes of the neuron model, the mean-driven and the excitable regimes and ask in all situations whether the presence of a strong periodic background stimulus can be beneficial for the detection task.

## Model and methods

### Population model and single-neuron model

We consider a population of spiking neurons that are not connected but driven by a common periodic signal and individual noise. In the example of weakly electric fish, the neurons would correspond to the P-units in the electric fish and the periodic input signal would contain components stemming from the nearby female fish and from the intruder to be detected. The scenario of a population of uncoupled noisy neurons that transmit information of periodic input stimuli is, however, more general and for instance also encountered in the auditory periphery.

The dynamics of the *i*-th spiking neuron is given by a leaky integrate-and-fire (LIF) model driven by an external signal *s*(*t*):

1 $$\begin{aligned} {\dot{v}}_{i}(t)&= -v_{i}(t) + \mu + \varepsilon s(t) + \sqrt{2 D} \xi _{i}(t), \\&\quad \text {with}\quad i = 1, \ldots , N_{\textrm{pop}}. \nonumber \end{aligned}$$

Here, $v_{i}(t)$ is the membrane voltage, $\mu$ is the mean input current, $\xi _{i}(t)$ is white Gaussian noise with zero mean $\left\langle \xi _{i}(t) \right\rangle = 0$ and correlation function $\left\langle \xi _{i}(t)\xi _{i}(t') \right\rangle = \delta (t-t')$ and *D* is the noise intensity. Whenever $v_{i}(t)$ hits the threshold $v_{T}$, a spike is registered for that time and $v_{i}(t)$ is reset to $v_{R}$. In our non-dimensional model, time is measured in units of the membrane time constant, the voltage in multiples of threshold-reset difference, and we set $v_{R} = 0$ and $v_{T} = 1$ (cf. [56]). In our setup, the sensory stimulus, common to all $N_{\textrm{pop}}$ units in the population, reads

2 $$\begin{aligned} s(t) = a_{s} \cos (\omega _{s} t + \varphi _{s}) + a_{b} \cos (\omega _{b} t + \varphi _{b}) \ . \end{aligned}$$

It is given by the sum of two cosine functions with different frequencies $\omega _{s,b} = 2\pi f_{s,b}$ (we will use both the regular frequencies $f_{s,b}$ and circular frequencies $\omega _{s,b}$), relative amplitudes $a_{s,b}$ and phase offsets $\varphi _{s,b}$; the total signal *s*(*t*) enters the dynamics scaled by a global amplitude $\varepsilon$. The two terms represent the total stimulus, consisting of a strong background stimulus $a_{b} \cos (\omega _{b} t + \varphi _{b})$ and the weak signal $a_{s} \cos (\omega _{s} t + \varphi _{s})$, the presence of which has to be detected from the output of the population. We will consider situations in which the weak signal is absent ($a_{s} = 0$) or present ($a_{s} > 0$) and will ask how the presence of this signal can be detected. We will also inspect how the detectability of the signal depends on the presence ($a_{b} > 0$) or absence ($a_{b} = 0$) of the background periodic stimulus.

For the numerical simulations of Eq. (1), we use the Euler–Maruyama method, operating in discrete time steps $t = t_{0} + \ell \Delta t$

3 $$\begin{aligned} v_{i}\bigl (t_{0} + (\ell +1) \Delta t \bigr )= & {} v_{i}(t_{0} + \ell \Delta t) \left( 1 - \Delta t\right) + \mu \Delta t \nonumber \\{} & {} + \varepsilon s(t_{0} + \ell \Delta t) \Delta t + \sqrt{2D \Delta t} \vartheta _{\ell } \ . \nonumber \\ \end{aligned}$$

Here, $\vartheta _{\ell }$ are independent Gaussian numbers drawn from a normal distribution with zero mean $\langle \vartheta _{\ell }\rangle = 0$ and unit variance $\left\langle \vartheta _{\ell } \vartheta _{k} \right\rangle = \delta _{\ell k}$. For all numerical examples in this paper, we use an integration time step of $\Delta t = 10^{-3}$.

We consider a neuron population of $N_{\textrm{pop}}= 10^{3}$ in all simulations. We analyze the time-dependent count statistics of the population for the different signal combinations in order to test a specific idea how the presence of the weak periodic signal may be detected (for details see below). To determine the time-dependent spike count *N*(*t*), we discretize the time axis into bins $\Delta t_{\textrm{bin}}= 0.05$ (significantly larger than our integration time step) and count all the spikes fired by all neurons within this bin:

4 $$\begin{aligned} N(t) = \sum _{i=1}^{N_{\textrm{pop}}} N_{i}[t, t + \Delta t_{\textrm{bin}}] \ . \end{aligned}$$

Dividing this by the size of $\Delta t_{\textrm{bin}}$ and an additional trial average yields an approximation of the instantaneous firing rate

5 $$\begin{aligned} r(t) \approx \sum _{i=1}^{N_{\textrm{pop}}} \frac{\left\langle N_{i}[t, t + \Delta t_{\textrm{bin}}] \right\rangle _{\xi _{i}}}{\Delta t_{\textrm{bin}}N_{\textrm{pop}}} \ , \end{aligned}$$

where $\left\langle \cdot \right\rangle _{\xi _{i}}$ indicates an ensemble average over realizations of the intrinsic noise $\xi _{i}(t)$ (the common signal is always the same in all these realizations).

### Detection process

Our approach here is similar in spirit to the recent study by Bernardi and Lindner [57] of the detection of a static signal embedded in an Ornstein–Uhlenbeck process; however, there are also important differences (see below).

**Fig. 1:**
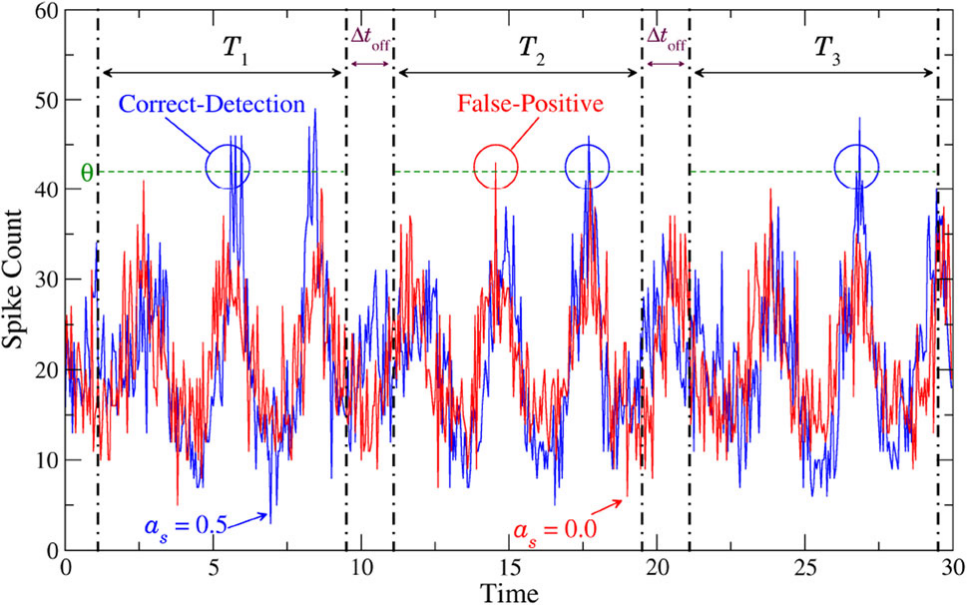
Illustration of the measurement process. One spike count modulation *N*(*t*) in the presence of the signal (blue) and one in absence of the signal (red) are shown as well as the first three detection windows. In all visible trials, the chosen threshold $\theta$ is exceeded by the blue trajectory implying a registration of a correct detection event for these trials. The red trajectory reaches $\theta$ only in $T_{2}$, i.e. we record a false-positive event for $T_{2}$ but not for $T_{1}$ or $T_{3}$. Remaining parameters: $a_{b} = 1.0$, $f_{s} = 0.1$, $f_{b} = 0.33$, $\varepsilon = 0.05$, $\Delta t_{\textrm{bin}}= 0.05$, $\mu =1.1$, $D=0.001$

Specifically, we will analyze two time series for different experiments, i.e., spike count modulations *N*(*t*) in the presence or absence of the signal. We will carry out this numerical experiment for the two distinct situations when the strong periodic background signal is present ($a_{b} = 1$ in Eq. (2)) or not ($a_{b} = 0$ in Eq. (2)). We measure the time-dependent counts in a very long time window that is split into $N_{T}$ smaller detection windows $T_{j}$ of length $T=K\Delta t_{\textrm{bin}}$ and short pauses of length $\Delta t_{\text {off}}$ cf (Fig. 1). The $N_{T}$ time windows serve as trials—this is somewhat different to the procedure in [57], where trials result from the repetition of the same experiment. At the same time the averaging over subsequent time windows implies an automatic averaging over the initial phases of the periodic signals (as long as the time window is not a multiple of one the driving signal’s period, a non-generic case that we exclude in the following by $\Delta t_{\text {off}}$).

We assume that the detection of an event takes place whenever the spike count of the population crosses a threshold $\theta$ (green dashed horizontal line in Fig. 1). In two distinct numerical simulations of our population model, the count in the presence (blue) and in the absence of the signal (red) is measured, respectively. When the blue time series crosses $\theta$ at least once within the corresponding time window, a correct-detection is registered for that trial. In analogy to this, a false-positive event is recorded when the red time series exceeds the threshold at least once. The correct detection (CD) and false-positive (FP) rates are then obtained by averaging over all $N_{T}$ trials. Varying the threshold yields the two rates as functions of $\theta$.

**Fig. 2:**
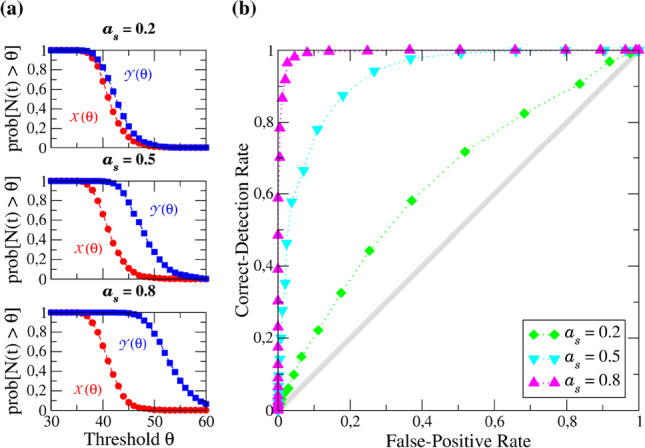
**a** FP rates ${\mathcal {X}}(\theta )$ and CD rates ${\mathcal {Y}}(\theta )$ obtained from simulations for different values of the signal amplitudes as indicated in each subplot title, **b** corresponding ROC curves. Remaining parameters: $a_{b} = 1$, same as in Fig. 1

In Fig. 2 a few examples for the FP and CD rates vs threshold $\theta$ for different values of the signal amplitude $a_{s}$ are shown in (a) together with the corresponding ROC curves in (b). The latter are obtained by plotting the CD rate as a function of the FP rate. A low $\theta$ corresponds to a very high detector sensitivity and is indicated by the upper right corner in Fig. 2b, and a high $\theta$ is represented by the lower left range. The example curves are taken for $a_{b} = 1$, which means in the presence of the strong periodic background stimulus.

We first focus on the CD (blue) and FP (red) rates as functions of the threshold $\theta$ in Fig. 2a. At small $\theta$, the probability of the measured spike count *N*(*t*) to be above the threshold at least once within the time window is close to one and decreases with increasing $\theta$ toward zero. For a weak signal, $a_{s} = 0.20$, the CD and FP rates are very close to each other implying that the detection of the signal is difficult. Boosting the signal amplitude leads to a higher spike count, thus the CD rate starts to decrease for higher threshold values, while the FP rate remains the same. As a consequence, the horizontal distance between the CD and FP rates is increasing for higher signal amplitudes and detection becomes a simpler task as can be expected. The improved detectability for larger $a_{s}$ is also apparent in the ROC curves in Fig. 2b. For a weak signal amplitude ($a_{s} = 0.2$, green), the distance to the diagonal (gray solid line, representing chance level) is small. Enlarging the signal amplitude yields a growing distance from the diagonal. In the present examples, the ROC curve of the signal amplitude $a_{s} = 0.8$ (pink) is already close to that of an ideal detector, i.e. a detector with a $100\%$ CD rate at all FP rates.

### Analytical approximation of the ROC

To derive an analytical description of the ROC curve, we make two assumptions for the count in a short time bin ($\Delta t_{\textrm{bin}}\ll \langle I\rangle$, where *I* denotes the interspike interval of a single stochastic LIF neuron):

1. The spike count distribution follows a Poisson distribution $P_{\langle N_{k}\rangle } (N)$;
2. spike counts in each time bin are independent.

The first assumption is well-justified for a sufficiently small time bin (see e.g. [58]). The second assumption is certainly an approximation, as the sum of independent non-Poissonian spike trains do not converge to a Poisson process [59]. Assuming a Poisson count statistics has the great advantage that we just need to know the mean spike count $\langle N_{k}\rangle$ to determine the distribution completely. The basic idea is identical to the procedure in the numerical case: We want to estimate the probability to be at least once in the detection window above a certain threshold $\theta$.

We first determine the probability for the spike count *N* in a time bin $\Delta t_{\textrm{bin}}$ to be below or at most at the threshold $\theta$. This is given by the sum over all possible values of $N \in {\mathbb {N}}$ up to $\lfloor \theta \rfloor$, the largest integer smaller than or equal to $\theta$:

6 $$\begin{aligned} \text {prob} \left( N \le \theta \right)= & {} \sum _{N = 0}^{\lfloor \theta \rfloor } P_{\langle N_{k}\rangle } (N) = e^{-\langle N_{k}\rangle } \sum _{N = 0}^{\lfloor \theta \rfloor } \frac{\langle N_{k}\rangle ^{N}}{N!} \nonumber \\= & {} \frac{\Gamma \left( 1 + \lfloor \theta \rfloor , \langle N_{k}\rangle \right) }{\lfloor \theta \rfloor !} \approx \frac{\Gamma \left( 1 + \theta , \langle N_{k}\rangle \right) }{\Gamma (1 + \theta )} \ . \nonumber \\ \end{aligned}$$

In the last line, we used the incomplete Gamma function $\Gamma (a,x) = \int _{x}^{\infty } \text {d} t \ t^{a-1} e^{-t}$ [60] and furthermore provided in the last step an approximate expression that interpolates between integer values of $\theta$ (at the latter, the two expressions coincide). In the following, we will use for simplicity only the latter expression.

To estimate the probability $p(\theta , T)$ to be not even once above $\theta$ in the *j*-th detection window $T_{j}$, we multiply the probabilities Eq. (6) from all bins, exploiting the assumption of statistical independence that holds true for a Poisson process:

7 $$\begin{aligned} p(\theta , T) = \prod _{k = 0}^{K-1} \frac{\Gamma \left( 1 + \theta , \langle N(t_{j;k})\rangle \right) }{\Gamma \left( 1 + \theta \right) } \ . \end{aligned}$$

Here, we have used the population spike counts at times $t_{j,k} = j(T + \Delta t_{\text {off}})+k\Delta t_{\textrm{bin}}$.

The FP ${\mathcal {X}}(\theta , 0, T)$ and CD ${\mathcal {Y}}(\theta , a_{s}, T)$ rates are then given by

8 $$\begin{aligned} {\mathcal {X}}(\theta , 0, T)= & {} \frac{1}{N_{T}} \sum _{j=0}^{N_{T}-1} \left( 1 - \Biggl . \right. \nonumber \\{} & {} \quad \left. \prod _{k\!=\!0}^{K-1} \frac{\Gamma \left( 1 \!+\! \theta , \left\langle N(t_{j,k}; a_{s} \!=\! 0) \right\rangle \right) }{\Gamma \left( 1\!+\!\theta \right) }\right) , \end{aligned}$$

9 $$\begin{aligned} {\mathcal {Y}}(\theta , a_{s}, T)= & {} \frac{1}{N_{T}} \sum _{j=0}^{N_{T}-1} \left( 1 - \Biggr . \right. \nonumber \\ {}{} & {} \quad \left. \prod _{k=0}^{K-1} \frac{\Gamma \left( 1\! +\! \theta , \left\langle N(t_{j,k}; a_{s} \!>\! 0) \right\rangle \right) }{\Gamma \left( 1 \!+\! \theta \right) }\right) , \end{aligned}$$

where we indicated the explicit dependence of the spike count on the signal amplitude by a parametric argument. Furthermore, the above formulas also include the trial average over the detection windows $T_{j}$ (sum over *j*).

With the obtained expression, we can proceed in two different ways. Firstly, we can measure the time-dependent mean spike count in simulations and use these data in Eqs. (8) and (9). This will be referred to as a semi-analytical theory in the following as it still requires some numerical simulations. Secondly, we can approximate the mean spike count using linear and nonlinear response theory for stochastic integrate-and-fire neurons that are driven by periodic signals [61, 62]. In the latter case, we will use Eq. (5) to estimate the mean spike count via

10 $$\begin{aligned} \langle N(t_{j;k})\rangle \approx r(t_{j;k}) \Delta t_{\textrm{bin}}N_{\textrm{pop}}\ . \end{aligned}$$

The instantaneous firing rate in the weakly nonlinear regime (neglecting higher than second-order terms in $\varepsilon$) can be approximated by [62]

11 $$\begin{aligned} r(t)&\approx r_{0} + \frac{\varepsilon ^{2} a_{s}^{2}}{2} \chi _{2} \left( \omega _{s}, -\omega _{s}\right) + \frac{\varepsilon ^{2} a_{b}^{2}}{2} \chi _{2} \left( \omega _{b}, -\omega _{b}\right) \nonumber \\&\quad + \varepsilon \Bigl [ a_{s} \left| \chi _{1}(\omega _{s}) \right| \cos \bigl ( \omega _{s} t + \varphi _{s} - \phi _{1}(\omega _{s}) \bigr ) \nonumber \\&\quad + a_{b} \left| \chi _{1}(\omega _{b}) \right| \cos \bigl ( \omega _{b} t + \varphi _{b} - \phi _{1}(\omega _{b}) \bigr ) \Bigr ]_{\text {LR}} \nonumber \\&\quad {+} \frac{\varepsilon ^{2}}{2} \Bigl [ a_{s}^{2} \left| \chi _{2} \left( \omega _{s}, \omega _{s}\right) \right| \cos \bigl ( 2 \omega _{s} t {+} 2 \varphi _{s} {-} \phi _{2} \left( \omega _{s}, \omega _{s}\right) \bigr ) \nonumber \\&\quad {+} a_{b}^{2} \left| \chi _{2} \left( \omega _{b}, \omega _{b} \right) \right| \cos \bigl ( 2\omega _{b} t {+} 2\varphi _{b} {-} \phi _{2} \left( \omega _{b}, \omega _{b}\right) \bigr ) \Bigr ]_{\text {HH}} \nonumber \\&\quad + \varepsilon ^{2} a_{s} a_{b} \Bigl [ \left| \chi _{2} \left( \omega _{s}, \omega _{b}\right) \right| \times \nonumber \\&\quad \cos \bigl ( \left( \omega _{s} + \omega _{b}\right) t + \varphi _{s} + \varphi _{b} - \phi _{2} \left( \omega _{s}, \omega _{b}\right) \bigr ) \Bigl . \nonumber \\&\quad \Bigr . + \left| \chi _{2} \left( \omega _{s}, -\omega _{b}\right) \right| \times \nonumber \\&\quad \cos \bigl ( \left( \omega _{s} - \omega _{b}\right) t + \varphi _{s} - \varphi _{b} - \phi _{2} \left( \omega _{s}, -\omega _{b}\right) \bigr ) \Bigr ]_{\text {MR}} \ . \end{aligned}$$

Here, we have included initial phases $\varphi _{s,b}$ for both periodic signals. The indices in the above expression indicate distinct contributions to the response: the steady state with an index 0, the linear response (LR), the higher harmonics of the periodic driving (HH) and the mixed response (MR) that emerges because of the simultaneous presence of two signals (see [62] for further discussion). All the firing and response characteristics for the white-noise-driven LIF model, $r_{0}, \chi _{1}(\omega ), \chi _{2}(\omega _{1},\omega _{2})$, are given in Appendix A.

The FP ${\mathcal {X}}_{\text {ana}}(\theta ,0,T)$ and CD ${\mathcal {Y}}_{\text {ana}}(\theta ,a_{s},T)$ rates for the analytical theory can then be expressed by

12 $$\begin{aligned} {\mathcal {X}}_{\text {ana}}&(\theta , 0, T) = \frac{1}{N_{T}} \sum _{j=0}^{N_{T}-1} \left( 1 - \Biggl . \right. \nonumber \\&\quad \left. \prod _{k=0}^{K-1} \frac{\Gamma \left( 1 + \theta , r(t_{j,k}; a_{s} = 0) \Delta t_{\textrm{bin}}N_{\textrm{pop}}\right) }{\Gamma \left( 1 + \theta \right) }\right) \ , \end{aligned}$$

13 $$\begin{aligned} {\mathcal {Y}}_{\text {ana}}&(\theta , a_{s}, T) = \frac{1}{N_{T}} \sum _{j=0}^{N_{T}-1} \left( 1 - \Biggr . \right. \nonumber \\&\quad \left. \prod _{k=0}^{K-1}\frac{\Gamma \left( 1 + \theta , r(t_{j,k}; a_{s} > 0) \Delta t_{\textrm{bin}}N_{\textrm{pop}}\right) }{\Gamma \left( 1 + \theta \right) }\right) \ . \end{aligned}$$

The FP rate in the absence of the strong periodic background stimulus ($a_{b} = 0$) can be simplified: In this case, we also have for the signal amplitude $a_{s} = 0$, so the instantaneous firing rate in Eq. (11) reduces to $r(t)\approx r_{0}$, and we obtain

14 $$\begin{aligned} {\mathcal {X}}_{\text {ana}}(\theta , T) = 1 - \left( \frac{\Gamma \left( 1 + \theta , r_{0} \Delta t_{\textrm{bin}}N_{\textrm{pop}}\right) }{\Gamma \left( 1 + \theta \right) }\right) ^{K}. \end{aligned}$$

## Results

In the following, we investigate the signal detection task in two very different parameter regimes of the LIF neurons: neurons of the population are either mean-driven ($\mu = 1.1 > v_{T}$, $D=0.001$) or in an excitable regime ($\mu = 0.9 < v_{T}$, $D = 0.005$). We will see that the weakly nonlinear response will have a very different impact in the two regimes. We consider variations of the detection and signal parameters for both regimes. We are particularly interested in how the presence of the strong background stimulus affects the detectability of the weak signal. Simulations were performed for a total number of $N_{\textrm{pop}}= 10^{3}$ LIF neurons, and to create the ROC curves, $N_{T} = 10^{3}$ detection windows have been used.

### Change of the detection time window

**Fig. 3:**
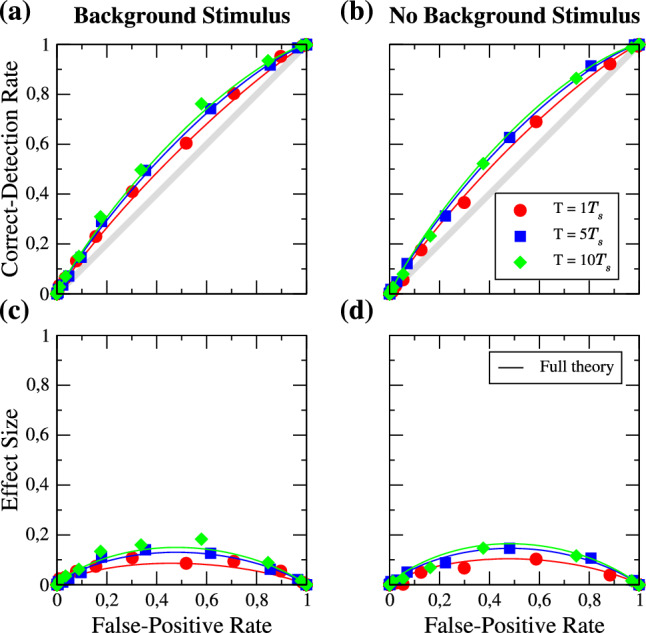
Excitable regime: ROC curves for different lengths of the detection window *T* in the presence (**a**) and absence (**b**) of the background stimulus. The length is given in multiples of the period of the signal $T_{s}$ as indicated in the legend. Symbols are numerical simulations, and solid lines represent the analytical theory of the ROC curves. **c**, **d** show the effect size as a function of the FP rate. Parameters: $f_{s} = 0.1$, $f_{b} = 0.33$, $a_{s} = 0.2$

Firstly, we would like to study the impact of the detection time window *T* in the excitable regime. Figure 3 shows the ROC curves for a relatively weak signal ($a_{s} = 0.2$), in the presence (Fig. 3a, $a_{b} = 1$) and absence (Fig. 3b, $a_{b} = 0$) of the strong periodic background stimulus. Figure 3c, d show the differences of the CD and FP rates as a function of the FP rate, which is referred to as the *effect size* [63, 64]. The different values of *T* are given in multiples of the period of the signal $T_{s}$ and are represented by different symbols and colors as indicated in the legend. The solid lines represent the analytical theory for the ROC curves (Eq. (13) plotted vs Eq. (12)). Three observations can be made. First of all, for the parameters chosen, the theory is in good agreement with the simulation results. Secondly, the effect of increasing the time window is very weak: a tenfold increase in the detection time window does not even lead to a doubling of the effect size, i.e. in the detectability of the signal. Thirdly, there is not much of a difference in the detectability introduced by the presence of the background stimulus.

**Fig. 4:**
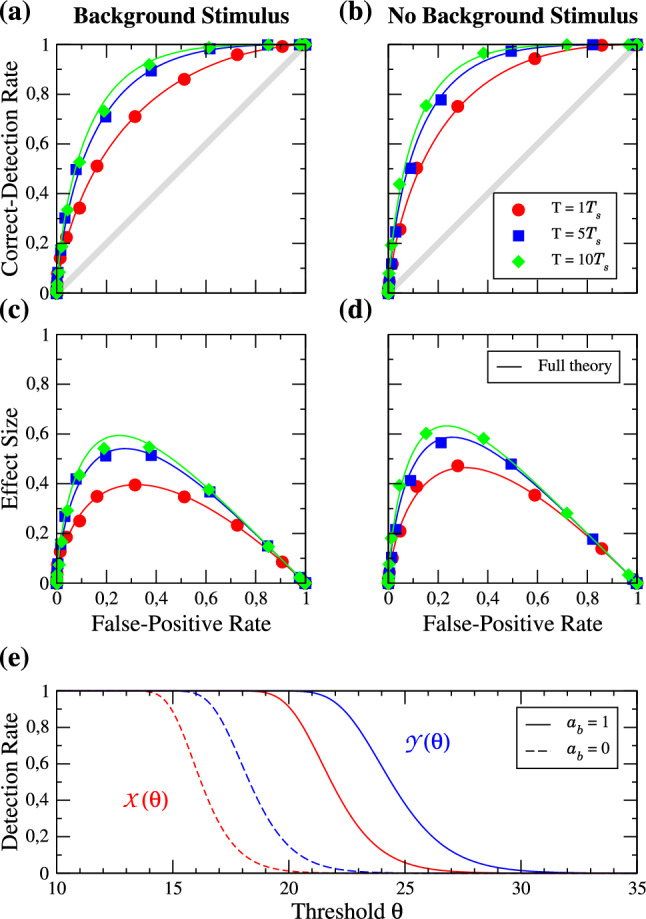
**a**–**d** Same as Fig. 3 but for an signal amplitude of $a_{s} = 0.5$, **e** FP (red) and CD (blue) rates in presence (solid lines) and absence (dashed lines) of the background stimulus

Next, we look at the same statistics for a larger signal amplitude (cf. Fig. 4); the general effect of $a_{s}$ will be inspected in the next subsection. The effect size is generally larger than before, i.e. the ROC curves are further away from the diagonal (see panels (a), (b)). The full analytical theory still works and the effect of enlarging the time window is still weak. If we compare the detection in the presence and in the absence of the background stimulus, we find that its presence can diminish the detection performance slightly. In panel (e) we show the FP and CD rates as functions of the threshold in the absence and the presence of the background stimulus for the detection window of $T=5T_{s}$; the main effect of the background stimulus is to shift the rates to higher thresholds, which has no effect on the ROC curves.

**Fig. 5:**
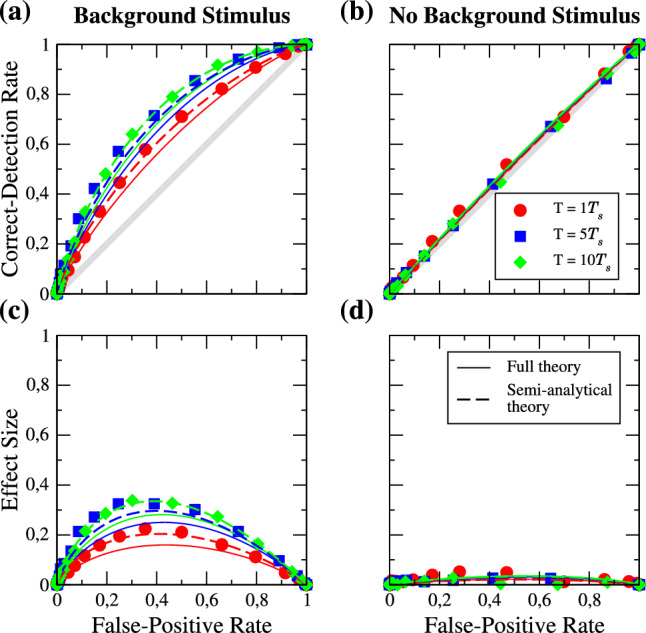
Mean-driven regime: ROC curves for different lengths of the detection window *T* in the presence (**a**) and absence (**b**) of the background stimulus. The length is given in multiples of the period of the signal $T_{s}$ as indicated in the legend. Symbols are numerical simulations, solid lines represent the analytical theory, and dashed lines the semi-analytical theory of the ROC curves. **c**, **d** show the effect size as a function of the FP rate. Parameters: $f_{s} = 0.1$, $f_{b} = 0.33$, $a_{s} = 0.2$

We now turn to the mean-driven regime. The ROC curves and corresponding effect sizes in the presence and absence of the strong background stimulus for a signal amplitude of $a_{s} = 0.2$ are shown in Fig. 5. In marked contrast to the excitable case, we find that the detector benefits from the presence of the background stimulus; there is a significant boost in the detectability of the weak periodic signal going from panel (d) to panel (c). Indeed, without the background stimulus, the ROC curves are practically on the diagonal and detection is nearly impossible.

Like in the excitable regime, the effect of enlarging the detection time window is weak. Furthermore, we notice that the analytical theory (solid lines) differs somewhat from the simulation results. This begs the question which part of our approximate calculation is responsible for this deviation. In order to answer this, we have determined by many simulations of the same periodic stimulus the time-dependent mean spike count that is the crucial input to the semi-analytical theory, Eq. (8), and Eq. (9). If we use these simulated mean count data (dashed lines in Fig. 5), the agreement is again very good. This means that the noticeable deviations of the full theory are due to the limitations of the weakly nonlinear response theory: taking only the terms up to the second-order in $\varepsilon$ does not reproduce the firing rate correctly. To obtain a better approximation of the firing rate and therefore also a good agreement of the analytical theory with the simulation results, one has to take higher order terms into account (see [65] for such a computation).

**Fig. 6:**
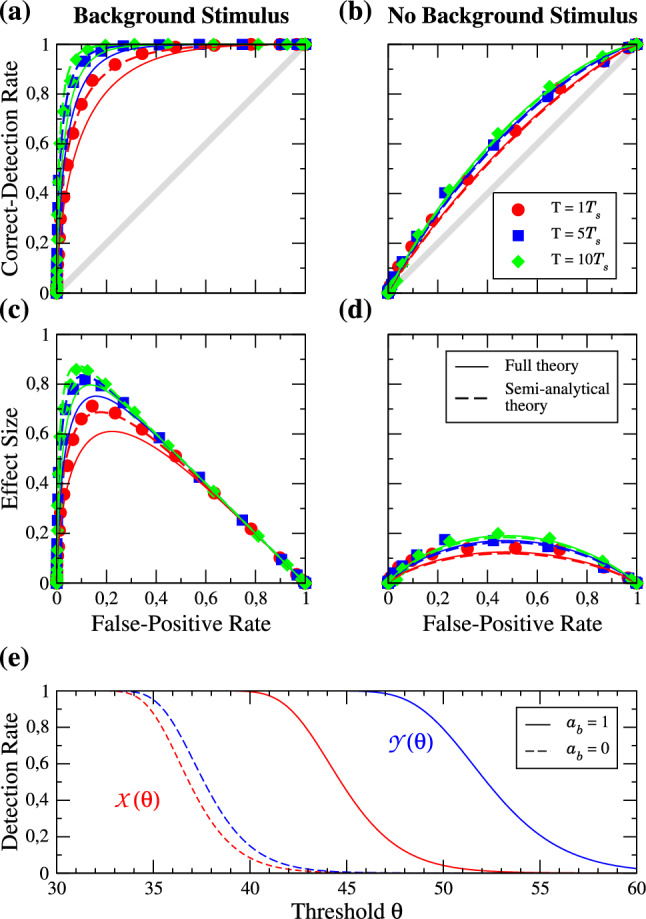
**a**–**d** Same as Fig. 5 but for an signal amplitude of $a_{s} = 0.5$, **e** FP (red) and CD (blue) rates in presence (solid lines) and absence (dashed lines) of the background stimulus

Next, we look at the effect of using a larger signal amplitude in the mean-driven regime (cf. Fig. 6). As in the excitable regime, the effect size is generally larger than before. We again find that the analytical theory differs noticeable from the simulation results but that the use of the numerically determined mean spike counts restores a good agreement. We also note that in the absence of the background stimulus, our full analytical theory works well.

Additionally, we show in Fig. 6e the FP and CD rates as functions of the threshold. In contrast to the excitable case, in the presence of the background stimulus ($a_{b} > 0$), the two rates are clearly further apart which improves detectability.

### Dependence on the signal amplitude

**Fig. 7:**
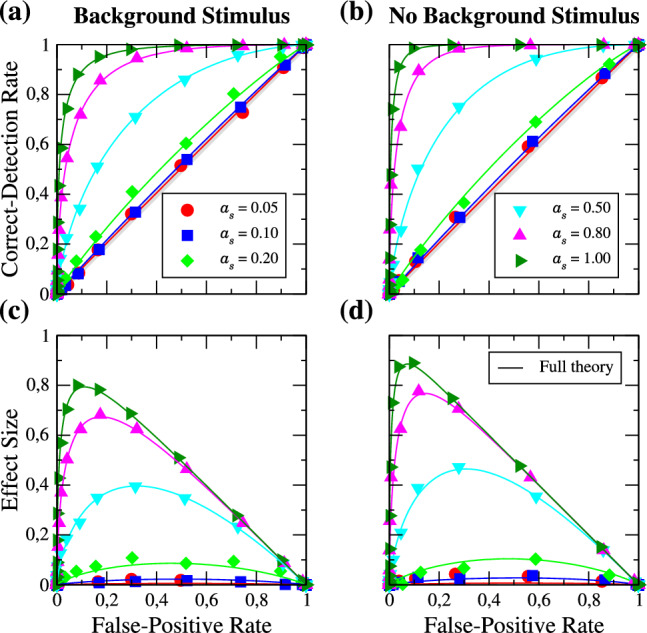
Excitable regime: ROC curves for different strengths of the signal amplitude $a_{s}$ in the presence (**a**) and absence (**b**) of the background stimulus. **c**, **d** show the effect size as a function of the FP rate. Parameters: $f_{s} = 0.1$, $f_{b} = 0.33$, $T = 10$

The detection problem is, obviously, particularly interesting for weak to moderate signal amplitudes, which we now explore in more detail. We again start with the excitable regime and consider in Fig. 7 the ROC curves for a range of amplitudes.

Referring to our three observations of the first subsection, we firstly notice that the full analytical theory is still in good agreement with our simulation results for all values of $a_{s}$ in the presence and absence of the background stimulus. Secondly, increasing the signal amplitude has a strong effect on the detectability, which is not surprising. But in contrast to enlarging the detection time window, a doubling of the value of $a_{s}$ may also lead to a doubling in the effect size in both cases (see panels (c) and (d)). Thirdly, for weak signal amplitudes ($a_{s} \le 0.2$) we observe again not much of a difference induced by the presence of the background stimulus. For relatively strong values of $a_{s}$, i.e. $a_{s} \ge 0.5$, the detectability of the signal seems to be better in the absence of the background stimulus, indicated by an up to $10\%$ higher effect size.

**Fig. 8:**
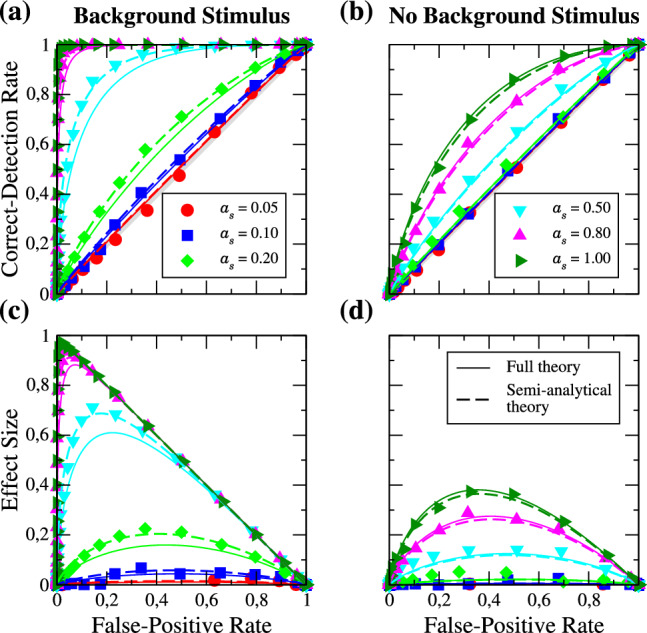
Same as Fig. 7 but in the mean-driven regime

In the mean-driven regime, cf. Fig. 8, we have a clear beneficial role of the background stimulus in the detection task. The deviations of the analytical theory to our simulation results in presence of the background stimulus are small and using the numerically determined mean spike count (semi-analytical approach) leads again to an excellent agreement.

**Fig. 9:**
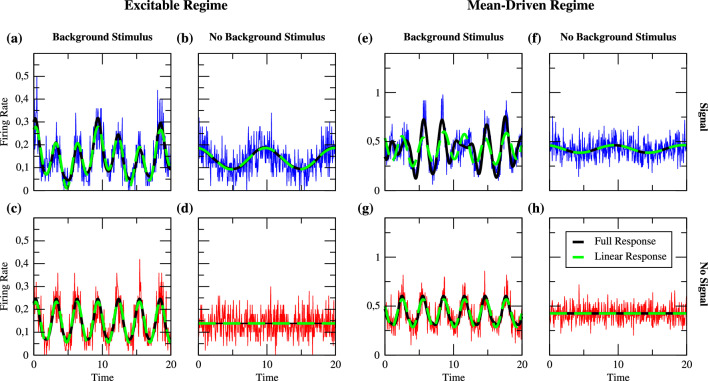
Firing rates obtained from simulations in the presence (blue) and absence (red) of the signal and also the strong background stimulus in the excitable (**a**–**d**) and mean-driven (**e**–**h**) regime. Black solid lines show the nonlinear response of the firing rate, green dashed lines the linear response

How does the beneficial effect of the background stimulus come about? To answer this question, we take a closer look at the firing rate in Eq. (11), especially at the influence of response terms of the first (linear response) and second order (higher harmonics and mixed response). Figure 9 shows the numerically obtained firing rate with and without the signal, with and without the background stimulus and in both excitable and deterministic firing regimes. In all panels, we also plot the full response up to the second order of the firing rate (solid black lines) as well as the response of the firing rate up to the linear order (dashed green lines).

We find, at least for the chosen frequencies, that the nonlinear response leads to a modestly increased rate modulation in the excitable regime if both background stimulus and signal are present (see panel (a)), whereas the nonlinear response has little effect on the rate in the absence of the signal. In marked contrast to this, in the mean-driven regime, we observe a strong boost of the firing rate modulation by the nonlinear response (see the pronounced difference between the black and green lines in panel (e)). A stronger rate modulation in the presence of the signal and the background stimulus plausibly enhances the detectability of the signal. Closer inspection of the single contributions of the nonlinear response reveals that the mixed response (MR term in Eq. (11)) is responsible for the beneficial boost of the rate modulation.

### Change of the frequencies of the periodic signals

So far, we have used a fixed combination of the frequencies of the two periodic signals. Now, we investigate how the detectability of the signal depends on these frequencies.

**Fig. 10:**
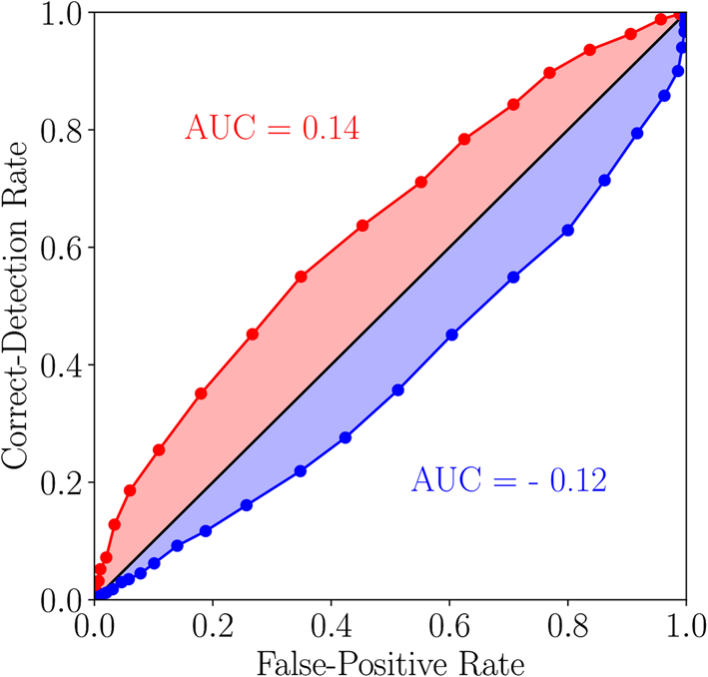
Visualization of the measure of detection performance. ROC curves above the diagonal will lead to a positive AUC value, below to a negative one

For a better visualization we use as a measure of detection performance the *area under the curve* (AUC): For each frequency combination, we measure the area enclosed by the ROC curve and the diagonal (c.f Fig. 10). If for instance the ROC curve falls on the diagonal, the performance measure is zero and detection is impossible. Systematic deviations from the diagonal indicate detectability beyond the chance level.

**Fig. 11:**
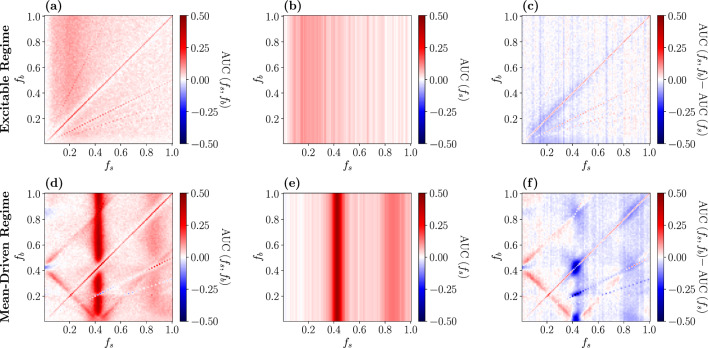
AUC measure in the excitable (**a**–**c**) and mean-driven regime (**d**–**f**) for ROC curves obtained from simulations. **a** and **d** in presence of the strong background stimulus, **b** and **e** in its absence, **c** and **f** show the beneficial effect of the strong background stimulus (red areas). Note that the colorbar in **c** and **f** is restricted to make the red areas more visible

The AUC measure allows us to identify conditions under which the presence of the background stimulus is beneficial for the detection of the weak periodic signal. We investigate this first for a comparatively weak signal ($a_{s} = 0.2$) and in the excitable regime, see Fig. 11a–c. We subtract the performance in the absence of the background stimulus (panel (b)) from the performance in its presence (panel (a)); the difference is shown in panel (c). First of all, the detectability seems to be best for a slow signal (see reddish part on the left in panel (a)); generally, the detectability is not very high in the excitable case for all frequency combinations. It is somewhat better if the background stimulus is absent (panel (b)), and the difference between the two cases (panel (c)) is either close to zero or slightly negative (vertical stripes and scattered blue and red points in panel (c) are measurement artifacts). This coincides with the special case investigated above: for neurons in the excitable regime, the presence of a strong background stimulus is detrimental for the detection of a faint signal.

In the mean-driven regime (c.f Fig. 11d–f), the situation becomes more interesting. Turning first to the case where the background stimulus is absent (see panel (e)), we observe a nearly perfect detection of the signal for a frequency of $f_{s} = 0.42$ and find a good performance for close-by frequencies (note the red vertical stripe around this frequency). The spontaneous firing rate of the neuron population with the used parameters (mean input $\mu$ and noise intensity *D*, see Eq. (A2)) is $r_{0} \approx 0.42$, so the detectability can be highly improved when the frequency of the driving signal either matches or is close to the spontaneous firing rate of the LIF neurons. Furthermore, the detection performance increases also for $f_{s} \approx 2r_{0}$ but the effect is much weaker (note the fainter red vertical stripe around $f_{s} = 2r_{0}$).

In the presence of the background stimulus (panel (d)), we also have an excellent detection of the weak signal for a signal frequency of $f_{s} \approx r_{0}$ and for almost all frequencies of the background stimulus (again, there is a pronounced vertical stripe around this frequency). However, much more striking is the improved detectability on certain diagonal lines on which either $f_{s} + f_{b} \approx r_{0}$ or $\left| f_{s} - f_{b}\right| \approx r_{0}$. These contributions arise due to the weakly nonlinear response of the neurons in the considered dynamical regime. The sum and difference of the two frequencies appear as contributions in the mixed response in Eq. (11) and lead to the beneficial role of the background stimulus in the detection of the faint signal, which becomes clear when considering the difference of the detectability measure in the presence and absence of the background stimulus (red areas in panel (f)). We note that in the motivating example of intruder detection in the courtship situation of weakly electric fish, the detection benefits for the behaviorally relevant frequency combinations, i.e. for $f_{s} < f_{b}$ and for $f_{s} \lessapprox r_{0}$—conditions that are a consequence of the distinct distributions of self-generated frequencies for male and female weakly electric fish [53] ($f_{s}$ and $f_{b}$ are the beating frequencies, i.e. they result from the difference of self-generated frequencies of interacting fish).

**Fig. 12:**
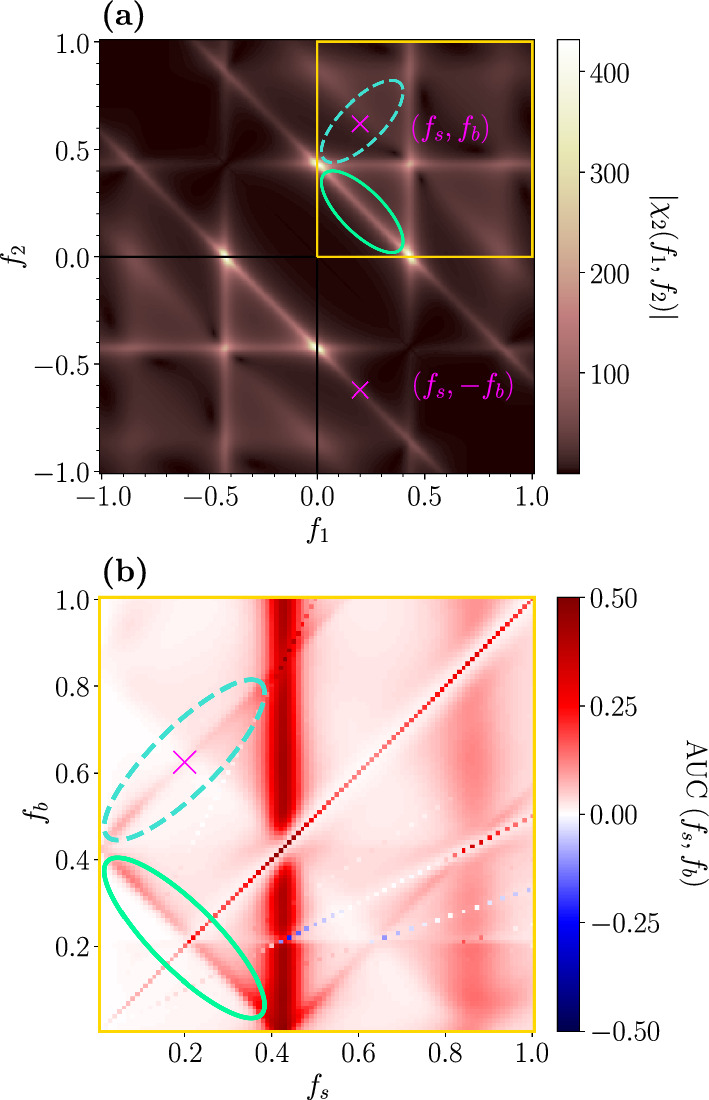
**a** Absolute value of the second-order susceptibility $\chi _{2}(f_{1},f_{2})$, **b** AUC measure in the presence of the background stimulus in the mean-driven regime for ROC curves obtained from weakly nonlinear response theory

Last we want to address the question, how the detection performance and the nonlinear response, especially the second-order susceptibility $\chi _{2}(f_{1},f_{2})$, are related. For this purpose, we use the analytical expression for $\chi _{2}(f_{1},f_{2})$ in combination with the expression for the firing rate modulation and our theory (Eq. (12) and Eq. (13)) to compute ROC curves for a broad range of frequency combinations $f_{1}, f_{2}$. Figure 12 shows the absolute value of the second-order susceptibility (panel (a)) and the AUC measure of the analytically obtained ROC curves (panel (b)).

Firstly, we observe a very good agreement of the analytically determined AUC measure (Fig. 12b) with the numerically obtained one (Fig. 11d). The small deviations of our analytical theory from the simulation results in the ROC curves found in the previous sections result in slightly different AUC values for some frequency combinations; however, the general structure of the detection performance is the same. Secondly, the absolute value of $\chi _{2}(f_{1}, f_{2})$ has some symmetry properties (for a detailed discussion, see [62]) which the AUC does not share due to the unequal roles of the two frequencies $f_{s}$ and $f_{b}$. Despite this lack of complete symmetry in the AUC, there are still similarities but also characteristic differences to $\left| \chi _{2}(f_{1},f_{2})\right|$. The most prominent similarities are the strong maximum around $f_{1} = r_{0}$ ($f_{s} = r_{0}$) and the other maximum around the antidiagonal line (highlighted by a green ellipse in Fig. 12 both in (a) and (b)) for which $f_{1} + f_{2} = r_{0}$ ($f_{s} + f_{b} = r_{0}$). The most striking differences are that $\left| \chi _2(f_{1}, f_{2})\right|$ is large around the horizontal line $f_{2} = r_{0}$, where the AUC is small and that the AUC is large around the two diagonals on which $\left| f_{s} - f_{b}\right| = r_{0}$ (one of them emphasized by a dashed turquoise ellipse in panel (b)) where the nonlinear response is very small (dashed blue ellipse in panel (a)). The difference between the AUC on the vertical line $f_{s} = r_{0}$ and the horizontal line $f_{b} = r_{0}$ is clear evidence of the unequal roles of the two frequencies. To understand why on the horizontal line the detection performance is not good it suffices to take into account that for $f_{b}$ close to the $r_{0}$, the firing rate is strongly modulated both in presence and absence of the signal, which makes it harder to detect a faint signal.

How can we explain the diagonal lines around $\left| f_{s} - f_{b}\right| = r_{0}$ that appear in panel (b) but not in panel (a) for $f_{1}, f_{2} > 0$ ($f_{s}, f_{b} > 0$)? Turning once again to Eq. (11), we note that the mixed response consists of two parts: (i) a term proportional to $\left| \chi _{2}(f_{s}, f_{b})\right|$—this contribution is strong when the condition $f_{s} + f_{b} \approx r_{0}$ is fulfilled (antidiagonal highlighted by the green ellipses); (ii) a term proportional to $\left| \chi _{2}(f_{s}, -f_{b})\right|$. The latter is just the mirrored diagonal line around the $f_{1}-$axis of $\left| \chi _{2}(f_{1}, f_{2})\right|$ (compare the dashed turquoise to the solid turquoise ellipse), where the absolute value of the second-order susceptibility is again comparably high. This leads to a strong mixed response not only for frequency combinations around $f_{s} + f_{b} = r_{0}$ but also around $\left| f_{s} - f_{b}\right| = r_{0}$ in the firing rate modulation and thus in the AUC.

## Summary and conclusions

In this paper, we considered a homogeneous population of LIF neurons driven by two periodic stimuli, one playing the role of a strong periodic background and the other one that of a faint signal to be detected. We investigated the detection performance in the excitable and mean-driven regimes and varied the signal and detection parameters. We developed an analytical framework to calculate ROC curves for the population activity approximately and demonstrated that our formulas which use the linear and the weakly nonlinear response of the instantaneous firing rate work reasonably well.

In general, we found in both regimes that the effect of enlarging the detection time window is very weak, whereas increasing the signal amplitude leads to a strong improvement in the detectability of the faint signal. For specific frequency combinations and in the mean-driven regime, the detection benefits greatly from the presence of the background stimulus: the detectability of the weak signal increases for $a_{b} > 0$. We showed that this effect can be traced back to the weakly nonlinear response and in particular to the so-called mixed response to two periodic signals. In marked contrast, in the excitable case, the presence of the background stimulus was only detrimental to the detection of a faint signal.

The beneficial effect of the background periodic stimulus bears some resemblance to *vibrational resonance*, an effect observed in bistable [66] and excitable systems [67]: a high-frequency (HF) signal can assist the transmission of a weak low-frequency signal. Apart from the occurrence of two periodic drivings, there are however important differences of our problem to the setting in which a vibrational resonance can be obtained. We consider the detection and not the transmission of the weak periodic signal and use all kinds of thresholds when calculating the ROC curve. Vibrational resonance (similar to stochastic resonance) is considered for systems with an inherent threshold, and the resonance effect relies on a suboptimal setting of this threshold. The beneficial effect that we have found is not due to such a suboptimal threshold but, as highlighted above, due to the weakly nonlinear response of the single units in our population model.

With respect to our motivating example, the intruder detection in a courtship situation of weakly electric fish, our modeling assumptions still have severe limitations. Firstly, we assumed a homogenous population of LIF neurons, although encoding populations in the sensory periphery display a pronounced heterogeneity with respect to mean activity (distributions of firing rates are broad) and to variability (also the CV is broadly distributed). For instance, the P-units in weakly electric fish possess firing rates between 50–400 Hz and CV’s between 0.2$--$0.9 [68]. Furthermore, many neurons in the sensory periphery display a pronounced firing rate adaptation [69, 70] that can be incorporated in the integrate-and-fire framework with additional variables (see e.g. [71–75]). It is unclear how neural heterogeneity and adaptation will affect the detectability of a weak stimulus. These are exciting problems for future research.

## Data Availability

The datasets generated and analyzed during the current study are available from the corresponding author on reasonable request.

## Appendix A: Weakly nonlinear response theory

We briefly state the important measures needed for the applied model (for details see [62]). Note that we here consider the LIF model with white noise for a vanishing refractory period, which simplifies the analytical results given in the following somewhat.

The response of the firing rate *r*(*t*) to a weak signal $\varepsilon s(t)$ $(\varepsilon \ll 1)$ can be approximated by the first terms of a Volterra series

A1 $$\begin{aligned} r(t)&= r_{0} + \varepsilon \int \hbox {d}{t_{1}'} \ K_{1} (t_{1}') s(t-t_{1}') \nonumber \\&\quad + \varepsilon ^{2} \int \hbox {d}{t_{2}'} \int \hbox {d}{t_{2}''} \ K_{2}(t_{2}', t_{2}'') s(t-t_{2}') s(t-t_{2}'') \nonumber \\&\quad + {\mathcal {O}}(\varepsilon ^{3}) \ . \end{aligned}$$

Here, $r_{0}$ is the spontaneous firing rate, i.e. if no signal is present ($\varepsilon = 0$), $K_{1}$ and $K_{2}$ are the first-order and second-order response kernels. For the white-noise-driven LIF model, $r_{0}$ is given by [76]

A2 $$\begin{aligned} r_{0} = \left[ \sqrt{\pi } \int _{\frac{\mu - v_{T}}{\sqrt{2D}}}^{\frac{\mu - v_{R}}{\sqrt{2D}}} \hbox {d}{x}\ e^{x^{2}} \text {erfc}(x) \right] ^{-1} \ . \end{aligned}$$

In the frequency domain, Eq. (A1) reads

A3 $$\begin{aligned} {\tilde{r}}(\omega )&= r_{0} \delta (\omega ) + \varepsilon \chi _{1}(\omega ) {\tilde{s}}(\omega ) \nonumber \\&\quad + \varepsilon ^{2} \int \hbox {d}{\omega '} \ \chi _{2}(\omega -\omega ', \omega ') {\tilde{s}}(\omega -\omega ') {\tilde{s}}(\omega ') \nonumber \\&\quad + {\mathcal {O}}(\varepsilon ^{3}) \ , \end{aligned}$$

where $\chi _{1}$ and $\chi _{2}$ are the Fourier transforms of the respective kernels

A4 $$\begin{aligned} \chi _{1}(\omega )&= \int \hbox {d}{t} \ e^{i\omega t)}K_{1}(t) \ , \end{aligned}$$

A5 $$\begin{aligned} \chi _{2}(\omega _{1},\omega _{2})&= \iint \hbox {d}{t_{1}} \hbox {d}{t_{2}} \ e^{i\omega _{1} t_{1}} e^{i\omega _{2}t_{2}} K_{2}(t_{1},t_{2}).\quad \end{aligned}$$

In terms of the parabolic cylinder functions ${\mathcal {D}}_{k}(x)$ [60], the linear response function $\chi _{1}$ [77] is given by

A6 $$\begin{aligned} \chi _{1}(\omega ) = \frac{r_{0} i \omega }{\sqrt{D}(i \omega - 1)} \frac{ {\mathcal {D}}_{i \omega - 1} \left( \frac{\mu - v_{T}}{\sqrt{D}}\right) - e^{\Delta } {\mathcal {D}}_{i \omega - 1} \left( \frac{\mu - v_{R}}{\sqrt{D}}\right) }{{\mathcal {D}}_{i \omega } \left( \frac{\mu - v_{T}}{\sqrt{D}}\right) - e^{\Delta }{\mathcal {D}}_{i \omega } \left( \frac{\mu - v_{R}}{\sqrt{D}}\right) } \end{aligned}$$

with $\Delta = \left[ v_{R}^{2} - v_{T}^{2} + 2\mu (v_{T} - v_{R})\right] /(4D)$; an equivalent expression in terms of hypergeometric functions was given by Brunel et al. [78] (for the equivalence of the expressions, see [79]). The second-order nonlinear response function $\chi _{2}$ has been calculated by Voronenko and Lindner [62]:

A7 $$\begin{aligned} \chi _{2}(\omega _{1},\omega _{2})&= \frac{ r_{0} (1 - i \omega _{1} - i \omega _{2}) (i \omega _{1} + i \omega _{2}) }{2D (i \omega _{1} - 1) (i \omega _{2} -1) } \times \nonumber \\&\quad \frac{ {\mathcal {D}}_{i \omega _{1} + i \omega _{2} - 2} \left( \frac{\mu - v_{T}}{\sqrt{D}}\right) - e^{\Delta } {\mathcal {D}}_{i \omega _{1} + i \omega _{2} - 2} \left( \frac{\mu - v_{R}}{\sqrt{D}}\right) }{{\mathcal {D}}_{i \omega _{1} + i \omega _{2}} \left( \frac{\mu - v_{T}}{\sqrt{D}}\right) - e^{\Delta } {\mathcal {D}}_{i \omega _{1} + i \omega _{2}} \left( \frac{\mu - v_{R}}{\sqrt{D}}\right) } \nonumber \\&\quad + \frac{i \omega _{1} + i \omega _{2}}{\sqrt{2D}} \frac{ \left( \frac{\chi _{1} (\omega _{1})}{i \omega _{2} - 1} + \frac{\chi _{1} (\omega _{2})}{i \omega _{1} - 1} \right) {\mathcal {D}}_{i \omega _{1} + i \omega _{2} - 1} \left( \frac{\mu - v_{T}}{\sqrt{D}}\right) }{{\mathcal {D}}_{i \omega _{1} \!+ i \omega _{2}} \left( \frac{\mu - v_{T}}{\sqrt{D}}\right) \!- e^{\Delta } {\mathcal {D}}_{i \omega _{1} +\! i \omega _{2}} \left( \frac{\mu - v_{R}}{\sqrt{D}}\right) } \nonumber \\&\quad - \frac{i \omega _{1} + i \omega _{2}}{\sqrt{2D}} \frac{ \left( \frac{\chi _{1} (\omega _{1})}{i \omega _{2} - 1} + \frac{\chi _{1} (\omega _{2})}{i \omega _{1} - 1} \right) e^{\Delta } {\mathcal {D}}_{i \omega _{1} + i \omega _{2} - 1} \left( \frac{\mu - v_{R}}{\sqrt{D}}\right) }{{\mathcal {D}}_{i \omega _{1} + i \omega _{2}} \left( \frac{\mu - v_{T}}{\sqrt{D}}\right) \!- e^{\Delta } {\mathcal {D}}_{i \omega _{1} \!+ i \omega _{2}} \left( \frac{\mu \!- v_{R}}{\sqrt{D}}\right) } . \end{aligned}$$
